# Precision Medicine in Inflammatory Bowel Diseases

**DOI:** 10.3389/fphar.2021.653924

**Published:** 2021-04-13

**Authors:** Irene Marafini, Giovanni Monteleone

**Affiliations:** Gastroenterology Unit, Department of Systems Medicine, University of Rome “Tor Vergata”, Rome, Italy

**Keywords:** crohn’s disease, ulcerative colitis, IBD, personalized medicine, anti-TNF

## Abstract

During the last decades, a better understanding of the mechanisms sustaining the pathogenic process in inflammatory bowel diseases (IBD) has contributed to expand the therapeutic armamentarium for patients with these disorders. Alongside with traditional therapies, monoclonal antibodies against tumor necrosis factor-α, the interleukin (IL)-12/IL-23 p40 subunit and the α4β7 integrin, and tofacitinib, a small molecule inhibiting intracellular pathways downstream to cytokine receptors, have entered into the clinic. However, these drugs are not effective in all patients and some responders can lose response over time. Such a therapeutic failure is, at least in part, dependent on the fact that, in IBD, the tissue damage is driven by simultaneous activation of multiple and distinct immune-inflammatory signals and the detrimental mucosal immune response changes over time even in the same patient. Therefore, personalized approaches aimed at identifying which patient should be treated with a specific drug at a precise time point are worth pursuing. A such approach has the advantage to improve efficacy of the drug and limit adverse reactions, thereby improving quality of the life of the patients and reducing costs. In this review, we summarize all the available evidence about the possible role of precision medicine in IBD.

## Introduction

Inflammatory bowel diseases (IBD) are chronic, disabling, immune-mediated disorders of the gastrointestinal tract encompassing two main clinical entities: Crohn's disease (CD) and ulcerative colitis (UC) (Abraham and Cho, 2009). Although the aetiology of IBD is unknown, it has been suggested that IBD-associated tissue damage process is induced by an exaggerated immune response against luminal antigens, which is favoured by genetic predisposition and environmental factors (Macdonald and Monteleone, 2005; Digby-Bell et al., 2020).

In the last decades, the possibility to collect mucosal samples from inflamed gut of IBD patients, the use of preclinical models of intestinal inflammation and the advent of sophisticated molecular technologies have led to a better understanding about the mechanisms by which the local immune response promotes gut damage. This progress has promoted the development of many pharmacological compounds, which can target key factors of the IBD-associated mucosal inflammation (Marafini et al., 2019). Among these, various anti-cytokine and anti-integrin blockers and small molecules inhibiting Janus kinases (JAK) are now available for the treatment of IBD (Neurath, 2017; Friedrich et al., 2019; Digby-Bell et al., 2020). However, these drugs are not effective in all patients and some responders can lose response over time. The reasons why blockade of major inflammatory pathways is not beneficial in some IBD patients remain unknown even though a considerable amount of work has been made to explain the different outcomes of biologic therapy in IBD. One possibility is that some drugs are effective only in phases of the disease characterized by enhanced production/function of the target, in line with the demonstration that the cytokine response differs among patients and even in the same patient during the disease course (Zorzi et al., 2013; Eftychi et al., 2019). It is, also, conceivable that suppression of a specific inflammatory pathway can, paradoxically, activate additional and distinct immune signals, which amplify the pathogenic process. This occurs, for instance, in patients receiving TNF blockers, in which neutralization of TNF function has been associated with induction of pathogenic T helper (Th)-17 cell responses (Schmitt et al., 2019). Independently of the basic mechanisms underlying such a therapeutic failure, the above observations suggest the need for criteria to stratify patients and to tailor drugs individually. As for now, the therapeutic decision is made by the physician upon critical evaluation of patient’s age, disease activity and behaviour, and previous therapies (Ding et al., 2016; Kopylov and Seidman, 2016). Some clinical and demographic characteristics, including age of the patients, smoking habit, penetrating and perianal CD or biochemical parameters (C-reactive protein and albumin), can help guide therapy, but fail to provide information on the preferred class of drugs to select. Therefore, personalized approaches aimed at identifying which patient should be treated with a specific drug at a precise time point are worth pursuing. This would have the advantage to improve efficacy of the drug and limit adverse reactions, thereby improving quality of the life of the patients and reducing costs. Here, we revise all the available evidence about the possible role of precision medicine in IBD (Table 1).

**TABLE 1:** Summary of the current evidence on precision medicine in IBD.

Field of investigation	Disease	Class of drug	Summary of evidence	References
*Molecular endoscopy*	CD	anti-TNF	TNF positive cells detected through endoscopic confocal laser endomicroscopy predict response to anti-TNF	Atreya et al. (2014)
	CD	anti-integrin	α4β7 positive cells detected through endoscopic confocal laser endomicroscopy predict response to vedolizumab	Rath et al. (2017)
*Transcriptomics*	UC	anti-TNF	Differentially expressed genes separated responders from non-responders to infliximab therapy	Arijs et al. (2009)
	CD, UC	anti-TNF	High levels of oncostatin M in the gut are associated with non-response to anti-TNF therapy	West et al. (2017)
	CD, UC	anti-TNF	The percentage of plasma cells in colon biopsies is a biomarker of failure to anti-TNF	Gaujoux et al. (2019)
	CD, UC	anti-TNF	Up-regulation of CCL7-CCR2 pathway and down-regulation of TREM1 is present in non-responders to anti-TNF	Gaujoux et al. (2019)
	CD, UC	anti-TNF	TREM1 is down-regulated in patients responsive to anti-TNF	Verstockt et al. (2019)
	UC	anti-integrin	Increased mucosal levels of granzyme a and integrin αE are associated with response to etrolizumab	Tew et al. (2016)
*Genetics*	UC	anti-TNF	Patients homozygous for high-risk IL-23R variants are more likely to respond to infliximab	Jurgens et al. (2010)
	CD	anti-TNF	Fas ligand CC or CT genotype is associated with a higher rate of clinical response to infliximab than the TT genotype	Hlavaty et al. (2005)
	CD	anti-TNF	Homozygous variants of the IBD5 locus are associated to infliximab unresponsiveness	Urcelay et al. (2005)
	Early onset IBD	haematopoietic stem cell transplantation	Mutations in IL-10RA and IL-10RB are associated with a better outcome	Kotlarz et al. (2012)
*Immunoprofiling*	CD	anti-IL23p19	Baseline serum concentrations of IL-22 predict response to anti-IL-23p19	Sands et al. (2017)
*Gut microbiome*	CD	anti-integrin	Roseburia inulinivorans and burkholderiales species are more abundant at baseline among patients responders to vedolizumab	Ananthakrishnan et al. (2017)

## Molecular Endoscopy

The use of molecular endoscopy is a revolutionary approach to predict response to therapy in IBD. During colonoscopy, fluorescent antibodies anti-TNF can be topically sprayed directly onto the diseased mucosa and endoscopic confocal laser endomicroscopy facilitates detection and quantification of mTNF-bearing mucosal cells. Atreya and colleagues demonstrated that CD patients with high number of mTNF positive cells in the colon had significantly higher short-term response rates (92%) at week 12 after subsequent anti-TNF therapy as compared to patients with a low number of mTNF positive cells (15%) (Atreya et al., 2014). Moreover, this clinical response was maintained for 1 year of follow-up and was associated with mucosal healing (Atreya et al., 2014). Promising results were also obtained by the same group in a subsequent study, in which, fluorescent antibodies assessing the number of α4β7-positive cells in inflamed gut of CD patients were used to predict response to Vedolizumab, an antibody targeting the α4β7 integrin (Rath et al., 2017). Two patients with pericryptal α4β7-positive cells in inflamed mucosa showed sustained clinical and endoscopic remission to subsequent Vedolizumab therapy, while no α4β7 positive cells were observed during *ex vivo* confocal laser endomicroscopy in 3 patients with CD unresponsive to vedolizumab (Rath et al., 2017).

Altogether, these observations suggest that the use of molecular imaging may predict therapeutic responses to biological treatment and can be exploited for precision medicine in CD. Validation in multicentre studies on larger cohorts of patients is needed before this approach can be adopted in clinical practice.

## Transcriptomics

In recent years, many studies have been performed to assess whether transcriptomics, the study of gene expression, can predict response to biologics in IBD. Arijs and co-workers compared pre-treatment colonic mucosal gene signature profiles between responders and non-responders to infliximab in a cohort of UC patients refractory to conventional treatment (Arijs et al., 2009). The authors found 212 probe sets differentially expressed between patients who subsequently responded to infliximab and those who did not. The top five differentially expressed genes separated responders from non-responders with 95% sensitivity and 85% specificity (Arijs et al., 2009). West and colleagues showed that high levels of oncostatin M (OSM), its receptor (OSMR) and the related transcriptional modules in inflamed gut of IBD patients were associated with non-response to anti-TNF therapy (West et al., 2017). Moreover, in preclinical models of IBD, genetic deletion or pharmacological blockade of OSM significantly attenuated colitis (West et al., 2017). Overall, these findings support the pathogenic role of OSM in the gut and suggest that unresponsiveness to anti-TNF may be related to the activation of alternative pathways of tissue damage. Gaujoux et al. analysed publicly available genome expression profiles of colon biopsy samples derived from different cohorts of patients with IBD (Gaujoux et al., 2019). The authors found that the percentage of plasma cells was a robust pre-treatment biomarker of failure to anti-TNF therapy. These results were validated in 2 independent cohorts of immune-stained colon biopsy samples, where a plasma cellular score from inflamed biopsies was predictive of non-response. Non-responders to anti-TNF exhibited also up-regulation of CCL7-CCR2 pathway and down-regulation of TREM1 (Gaujoux et al., 2019). However, conflicting results were published by Verstock et al. who found that levels of circulating TREM1 were down-regulated in both CD patients and UC patients responsive to anti-TNF (Verstockt et al., 2019). Factors accounting for such a discrepancy remain unknown even though differences could, at least in part, rely on the definition of responsiveness to anti-TNF adopted in these studies (i.e., clinical response vs. endoscopic response respectively). Transcriptomics were also used to predict therapeutic response to etrolizumab, a monoclonal antibody neutralising the β7 integrin subunit. In UC patients, increased mucosal levels of granzyme A and integrin αE were significantly higher at baseline in patients with subsequent response to etrolizumab (Tew et al., 2016).

## Genetics

More than 200 susceptibility genes have been identified in IBD population (Jostins et al., 2012; Liu et al., 2015; de Lange et al., 2017). Some of these genes have also been studied as possible predictors of response to biologic therapy. For example, patients homozygous for high-risk IL-23R variants were more likely to respond to infliximab therapy compared to patients bearing low-risk IL-23R variants (Jurgens et al., 2010). In a Belgian cohort of 287 consecutive patients treated with infliximab for refractory luminal (*n* = 204) or fistulizing (*n* = 83) CD, the Fas ligand −843 CC or CT genotype was associated with a higher rate of clinical response to infliximab than the TT genotype (Hlavaty et al., 2005). Many other loci were found to be predictive of anti-TNF therapy response. For example, the homozygous variants of the IBD5 locus was associated to infliximab unresponsiveness in CD, but not UC, patients (Urcelay et al., 2005).

Many studies have examined whether NOD2, the first and strongest susceptibility gene identified for CD (Cuthbert et al., 2002), is useful to predict response to therapy. Two studies failed to demonstrate a link between NOD2 expression and response to infliximab (Mascheretti et al., 2002; Vermeire et al., 2002). A subsequent metanalysis of 4 studies confirmed that NOD2 polymorphisms were not significantly associated with response to adalimumab or infliximab (Wang et al., 2016). More recently, it was shown that CD patients bearing polymorphisms in NOD2 had anti-TNF trough levels in the subtherapeutic range more frequently than patients without such a polymorphism (Schaffler et al., 2018).

Paediatric patients with very early onset of IBD represent a rare sub-group of IBD that develop the disease early in life due to the presence of monogenic defects (Glocker et al., 2009). In this subgroup, mutations in IL-10RA and IL-10RB were associated with a better outcome after haematopoietic stem cell transplantation (Kotlarz et al., 2012) compared to patients with epithelial gene defects (Uhlig and Muise, 2017).

Inflammasomes are multiprotein complexes of the innate immunity that contribute to the activation of inflammatory response (Sutterwala et al., 2007). Upon stimulation, the inflammasomes promote the maturation of the pro-inflammatory cytokines IL-1β and IL-18 (Opipari and Franchi, 2015). In a patient with a gain of function mutation in NLRC4 (a gene encoding for a protein activating the inflammasome) and developing early enterocolitis, there was an excessive production of IL-18. Notably, treatment of the patient with IL-18 blocker attenuated the ongoing intestinal inflammation (Canna et al., 2017).

These studies highlight the possibility to exploit genetic data to apply personalized therapeutic approaches.

## Immunoprofiling

The best example of the use of immunoprofiling to predict therapeutic response is represented by the discovery that baseline serum concentrations of IL-22 in CD predicted response to anti-IL-23p19 (Sands et al., 2017). IL-23 is produced by various immune cells, especially antigen presenting cells, and is a key cytokine for the maintenance and expansion of Th17 cells, which in turn, together with other cell types, are responsible for IL-22 production (Neurath, 2019). In a phase IIA, placebo-controlled study of 119 adults with moderately-to-severely active CD, patients taking MEDI 2070, an anti-IL-23/p19 antibody, had greater reductions in serum IL-22 levels than did patients receiving placebo. Baseline serum IL-22 concentrations with a median value of less than 15.6 pg/ml were associated with clinical response and remission rates similar to patients receiving placebo, while patients receiving MEDI2070 with levels over this threshold had an increased likelihood of clinical response and clinical remission at week 8 (Sands et al., 2017). Although this study suggests the attractive hypothesis that serum levels of IL-22 can be used as a biomarker to predict response to IL-23p19 inhibitors, larger validating cohorts are required to bring this knowledge into clinical practice.

## Gut Microbiome

The analysis of gut microbiota is another tool, which can be used to predict response to therapy. Ananthakrishnan and colleagues conducted a prospective study in 85 IBD patients initiating anti-integrin therapy with vedolizumab (Ananthakrishnan et al., 2017). α-diversity was significantly higher among CD patients achieving remission at week 14. Moreover, Roseburia inulinivorans and Burkholderiales species were more abundant at baseline among CD patients achieving remission at week 14. Thirteen pathways were significantly enriched in baseline samples from CD patients achieving remission. No statistically significant differences were observed in UC patients (Ananthakrishnan et al., 2017). These data suggest that microbial changes may be used as promising marker of response to biologic therapies.

## Discussion

Considering the continuous enrichment of IBD therapeutic armamentarium, a major challenge is represented by the validation of biomarkers that can be used in clinical practice to predict response to therapy. In fact, clinical trials and real-life studies indicate that response to therapy is highly heterogenous among patients. Thus, the strategy to give the right drug, to the right patient at the right time has become a great research interest in this field. Although individual biomarkers may be promising, the use of a multimodal analysis in which clinical, endoscopic, genetic, transcriptional and immunological data are combined together could build a truly personalized approach. In 2017, Barber and colleagues using a prospective registry, predicted the response of 359 CD patients to their first anti-TNF therapy using clinical and genetic parameters combined together (Barber et al., 2016). In another prospective inception cohort study of paediatric patients with newly diagnosed CD in the United States and Canada, genotypes, ileal gene expression, antimicrobial serology, and ileal, rectal, and faecal microbiota were assessed in order to create a risk model for disease complications and efficacy prediction of subsequent anti-TNF therapy (Kugathasan et al., 2017). This approach allowed a more precise risk stratification and a better selection of patients more likely to benefit from anti-TNF therapy. A similar approach was applied in a cohort study recruiting paediatric patients with newly diagnosed UC. RNA sequencing was used to define rectal gene expression before treatment, and 16S sequencing was used to characterise rectal and faecal microbiota. After adjusting for clinical predictors, an antimicrobial peptide gene signature together with the abundance of specific bacterial species (Ruminococcaceae and Sutterella) were associated with corticosteroid-free remission at week 52 and showed to be a promising tool to guide therapeutic decisions (Hyams et al., 2019).

However, precision medicine in IBD is still at its infancy. Most of the above-discussed studies were performed using small cohorts and at experimental level. None of these biomarkers has been validated and it is now ready to enter into clinical practice. Great economic resources are needed to make this step. The optimum would be to include the research of predictive biomarkers in clinical trial designs. Usually, in clinical trials, the target population is selected taking into account only clinical and demographic characteristics, with results that almost never overcome 50% of response. The capacity to include the tools provided by precision medicine for a more accurate patients’ selection would greatly improve both clinical and endoscopic response to therapy.

Another important aspect to be considered is the absolute need of independent validation cohorts due to the risk of bias in big data analysis. For instance, gene expression patterns of CD8^+^ T cells were initially reported to correlate with clinical outcomes of adult IBD patients (Lee et al., 2011). However, more recently, Gasparetto and colleagues were unable to validate the findings of an association between CD8^+^ T-cell gene transcription and disease outcome in IBD (Gasparetto et al., 2021).

**FIGURE 1:**
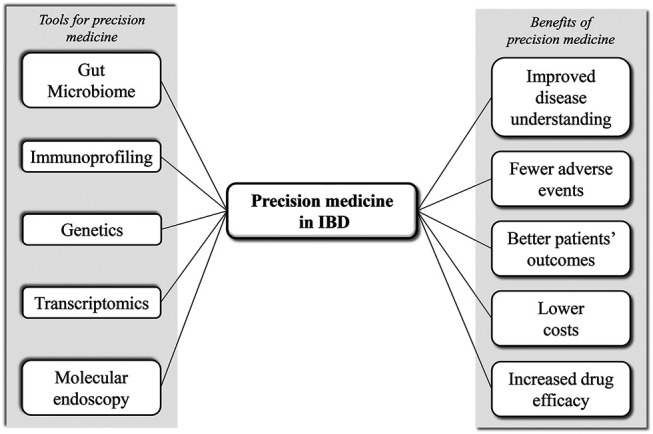
Schematic figure summarizing the tools and possible benefits of precision medicine in IBD.

An integrative personal profiling including all the tools for precision medicine (Figure 1), such as pharmacogenomics, gene expression profiling, proteomics (serum/tissues), metabolomics, immunoprofiling, microbiota analysis and imaging, can improve disease risk assessment, accuracy of diagnosis, disease monitoring and targeted treatments (Li-Pook-Than and Snyder, 2013). This is true for all the complex diseases, including IBD. Thus, we can imagine that, in the next future, a patient with a new diagnosis of IBD will undergo not only clinical, endoscopic and radiologic evaluation, but also transcriptomics, immunoprofiling and microbiota analysis. Altogether this information will be used to build-up a model for predicting individual risk and likelihood of response to specific therapies, with the potential to enable delivery of truly individualised IBD care.
